# How much allopurinol does it take to get to target urate? Comparison of actual dose with creatinine clearance-based dose

**DOI:** 10.1186/s13075-018-1755-0

**Published:** 2018-11-16

**Authors:** Lisa K. Stamp, Peter T. Chapman, Murray L. Barclay, Anne Horne, Christopher Frampton, Paul Tan, Jill Drake, Nicola Dalbeth

**Affiliations:** 1 Department of Medicine, University of Otago, Christchurch, P. O. Box 4345, Christchurch, 8140 New Zealand; 2 Department of Rheumatology, Immunology and Allergy, Christchurch Hospital, Private Bag 4710, Christchurch, 8140 New Zealand; 3 Department of Clinical Pharmacology, Christchurch Hospital, Private Bag 4710, Christchurch, 8140 New Zealand; 4 Department of Medicine, University of Auckland, Private Bag 92019, Auckland, New Zealand

## Abstract

**Objective:**

Allopurinol dosing has frequently been limited based on creatinine clearance (CrCL), resulting in failure to achieve target serum urate (SU). The aim of this analysis was to determine how many milligrams of allopurinol above the recommended CrCL-based dose (R+) are required to achieve target SU and to investigate the factors that influence R+.

**Methods:**

We analysed data from participants in a 24-month open, randomized, controlled, parallel-group, comparative clinical trial. Data obtained during the 12-month dose escalation (DE) phase of the study (year 1 for DE/DE and year 2 for control/DE) were combined. R+ dose was defined as the number of milligrams of allopurinol above the CrCL-based dose at the final visit.

**Results:**

Of the 132 participants, R+ allopurinol dose at the final visit was ≤ 100 mg/day in 38 (28.8%), 101–200 mg/day in 46 (34.8%) and > 200 mg/day in 48 participants (37.1%). There was no significant difference between the R+ groups in the number of participants achieving target SU. There was an increase in plasma oxypurinol and a larger percentage and absolute change in SU as R+ increased. Multivariate analysis revealed CrCL, weight, baseline SU and allopurinol dose, were significantly positively associated with allopurinol dose at 12 months. There were no significant differences across R+ groups in renal or liver function adverse events, although there were numerically more serious adverse events in the higher R+ groups.

**Conclusion:**

A wide range of R+ doses are required to achieve target SU. Four easily obtained clinical variables (baseline SU, CrCL, weight, and allopurinol dose) may be helpful to predict allopurinol dose.

**Trial registration:**

ANZCTR, ACTRN12611000845932. Registered on 10 August 2011.

**Electronic supplementary material:**

The online version of this article (10.1186/s13075-018-1755-0) contains supplementary material, which is available to authorized users.

## Introduction

Allopurinol remains the most commonly used urate-lowering therapy. Many clinicians have adhered to the creatinine-clearance (CrCL) based dosing recommendation published in 1984 by Hande et al. [1] believing that this will reduce the risk of the potentially fatal allopurinol hypersensitivity syndrome (AHS). However, while higher starting doses of allopurinol have been associated with AHS [2] there is little evidence that the maintenance dose, that is the dose of allopurinol required to achieve the widely recommended target urate [3, 4], is associated with AHS. Furthermore, a consequence of adopting a CrCL-based allopurinol dosing strategy is failure to achieve target serum urate (SU), with less than 20% of patients achieving SU < 6 mg/dL on such restricted doses [5]. We have recently published results of a randomized controlled trial showing that higher than CrCL-based allopurinol doses are safe and effective in people with gout [6, 7].

The aims of this pre-specified secondary analysis were to determine how much above CrCL-based allopurinol dose study participants required to achieve target SU, factors that influence the R+ requirement and identify any safety signal with higher R+ requirement.

## Methods

### Study design

A 24-month open, randomized, controlled, parallel-group, comparative clinical trial was undertaken (ACTRN12611000845932). Ethical approval was obtained from the Multi-Regional Ethics Committee, New Zealand. Written informed consent was obtained from each participant. Full methods have been reported previously [6, 7]. A total of 183 people with gout were randomized to continue current dose allopurinol for 12 months and then enter the dose escalation phase (control/DE) or to begin allopurinol dose escalation immediately (DE/DE). Allopurinol was increased monthly until SU was < 6 mg/dl. For those with CrCL ≤ 60 ml/min, allopurinol was increased by 50 mg increments and for those with CrCL > 60 ml/min by 100 mg. For the purposes of this analysis, data obtained during the dose escalation phase of the study (year 1 for DE/DE and year 2 for control/DE participants) were combined. Thus, baseline was month 0 for DE/DE and month 12 for control/DE participants. Six participants who discontinued allopurinol due to adverse events during the study and nine participants with no post-baseline values were excluded. A further 36 participants either died or were lost to follow up before they entered or during the dose escalation phase of the study. For the remaining 132 participants the CrCL-based allopurinol dose was calculated using baseline creatinine according to the Hande criteria [1]. R+ allopurinol dose was defined as the number of milligrams of allopurinol above baseline CrCL-based dose, and participants were grouped according to the allopurinol dose at the final visit of the dose escalation phase (i.e. month 12 for DE/DE and month 24 for control/DE) into one of the following three groups: R+ ≤ 100 mg/day, R+  101–200 mg/day, R+ ≥ 201 mg/day.

### Adverse and serious advent event reporting

Treatment emergent adverse events (AE) were defined as any AE occurring between month 0 and the end of month 12 in the DE/DE group and between month 12 and month 24 in the control/DE group. Worsening laboratory AEs were defined as those where there was an increase in AE grade using the Common Terminology Criteria for Adverse Events (v4.0) from the start of allopurinol dose escalation and the ensuing 12 months.

### Study outcomes

The primary efficacy outcome was mean serum urate at the end of the dose escalation phase (month 12 DE/DE or month 24 control/DE). Secondary efficacy outcomes included (1) the percentage reduction in SU at the final visit of the dose escalation phase, (2) actual reduction in SU at the final visit of the dose escalation phase and (3) the R+ dose of allopurinol required to achieve SU < 6 mg/dl. The primary safety outcomes were serious adverse events (SAEs) and treatment emergent or worsening liver or kidney function adverse events (AEs).

### Statistical analysis

Baseline demographic and clinical features were compared between R+ groups using one-way analysis of variance, the Wilcoxon rank test, and the chi^2^ test as appropriate. Comparisons between those with SU < 6 mmol/l and ≥ 6 mmol/l and those with or without concomitant diuretic within R+ groups were undertaken using one-way analysis of variance. Changes in SU and plasma oxypurinol were compared between R+ groups using one-way analysis of variance. Liver and renal function AE rates and SAEs were compared between R+ groups using the chi squared test. Multiple linear regression analysis was used to test the independent associations of baseline clinical features and dose of allopurinol at 12 months. A two-tailed *p* value <0.05 was taken to indicate statistical significance.

## Results

### Demographics

**Fig. 1:**
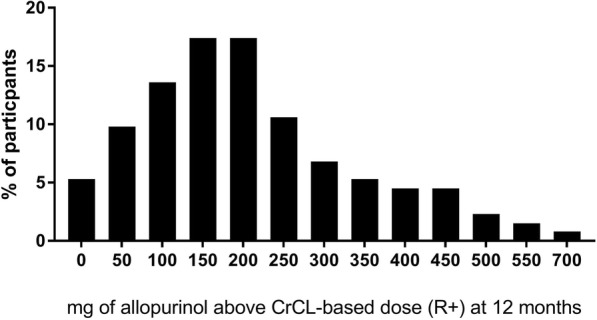
Amount of allopurinol (milligrams) above the recommended creatinine clearance (CrCL)-based dose (R+) dose at the end of the 12-month dose escalation phase of the study

Baseline demographics from the 132 participants who completed the 12-month dose escalation phase of the study are outlined in Table 1. Among these 132 participants, the R+ dose of allopurinol at the final visit was ≤ 100 mg/day in 38 (28.8%), 101–200 mg/day in 46 (34.8%) and > 200 mg/day in 48 participants (37.1%). Within these R+ dose groups there was a wide range of R+ doses, up to a maximum of 700 mg in the high-dose group (Fig. 1). Those who required R+ dose > 200 mg/day were significantly younger, had higher baseline SU, were more likely to be obese (body mass index ≥ 30 kg/m^2^) and were receiving more allopurinol at the start of the dose escalation phase (Table 1).

**Table 1:** Baseline clinical features and demographic features by R+ group as defined at month 12

	R+ ≤ 100 mg (*n* = 38)	R+ 101–200 mg (*n* = 46)	R+ > 200 mg (*n* = 48)	*P* value
Age, years^a^	61.2 (13.3)	64.1 (9.8)	55.1 (11.6)	0.001
Male, *n* (%)	32 (84.2)	40 (87.0)	46 (95.8)	0.18
NZ European, *n* (%)	20 (52.6)	25 (54.3)	16 (33.3)	0.08
Duration of gout years, median (range)	15.2 (1.2–40.4)	18.9 (0.16–49.5)	15.7 (0.37–47.4)	0.49
Baseline serum urate mg/dl^a^	6.6 (1.6)	6.6 (1.0)	7.6 (1.5)	<0.001
CrCl ml/min^a^	65.0 (29.4)	56.7 (19.4)	65.5 (27.9)	0.19
Weight kg^a^, mean (SD)	99.2 (21.6)	104.3 (21.4)	114.3 (20.6)	0.004
Body mass index, kg/m^2a^	32.2 (6.3)	35.2 (7.9)	36.6 (7.3)	0.23
Baseline allopurinol dose^a^	250 (103)	248 (67)	316 (123)	0.002
Baseline oxypurinol concentrations, μmol/l^a^	87.1 (42.0)	96.9 (47.9)	90.9 (56.8)	0.66
Co-existing conditions, *n* (%)				
Obesity^b^	20 (52.6)	35 (76.1)	41 (85.4)	0.003
Cardiovascular disease^c^	12 (31.6)	21 (45.7)	18 (37.5)	0.41
Diabetes mellitus	11 (28.9)	16 (34.8)	16 (33.3)	0.84
Hypertension	27 (71.1)	36 (78.3)	31 (64.6)	0.34
Hyperlipidemia	25 (65.8)	25 (54.3)	25 (52.1)	0.41
Diuretic, *n* (%)	12 (31.6)	22 (47.8)	18 (37.5)	0.30

*R+* amount of allopurinol (milligrams) above creatinine clearance-based dose, *CrCL* creatinine clearance

^a^Mean (SD)

^b^Obesity defined as body mass index ≥ 30 kg/m^2^

^c^CVD defined as ischemic heart disease, heart failure or peripheral vascular disease

### Serum urate

**Fig. 2:**
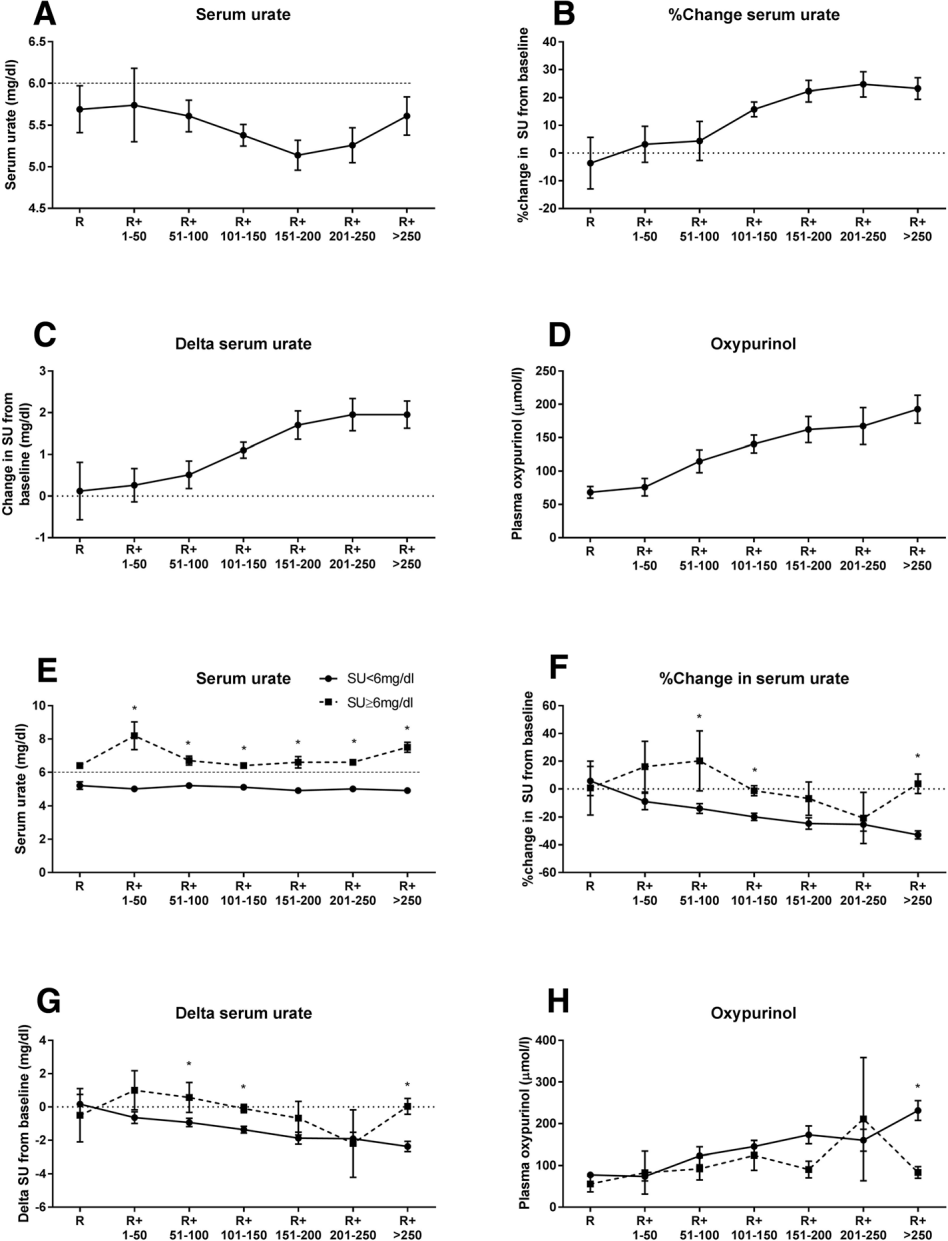
Effect of increasing the amount of allopurinol (milligrams) above the recommended creatinine-clearance-based dose (R+) on serum urate (SU) (**a**), percentage change in SU (**b**), delta SU (**c**) and plasma oxypurinol (**d**) at month 12 of the dose escalation phase. Difference in SU (**e**), percentage change in SU (**f**), delta SU (**g**) and plasma oxypurinol (**h**) at month 12 of the dose escalation phase in those who did or did not achieve target SU at month 12 of the dose escalation phase. *p < 0.05

There was no significant difference between the three R+ groups in the number of participants achieving target serum urate: ≤ 100 mg/day, 27/38 (71.1%); 101–200 mg/day, 38/46 (82.6%) and > 200 mg/day, 37/48 (77.1%); *p* = 0.45 (Table 2). There was an increase in plasma oxypurinol and a larger percentage change and absolute change in serum urate as the R+ increased (Fig. 2a–d and Table 2). There was a larger reduction in urate in those who achieved target urate versus those who did not (Fig. 2e–g). Plasma oxypurinol was not significantly different between those at target and those not at target except at the R+ 250 mg/day dose (Fig. 1h). There were no significant differences in mean serum urate, or percent and absolute change in SU in those receiving and not receiving a diuretic (Additional file 1: Figure S1). However, plasma oxypurinol was significantly higher in those receiving diuretics at the larger R+ doses. (Additional file 1: Figure S1).

**Table 2:** Changes in serum urate and plasma oxypurinol between R+ groups

	R+ ≤ 100 mg (*n* = 38)	R+ 101-200 mg (*n* = 46)	R+ > 200 mg (*n* = 48)	P
SU < 6 mg/dl, *n* (%)	27 (71.1)	38 (82.6)	37 (77.1)	0.45
Mean SU, mg/dl (mean (SEM))	5.7 (0.18)	5.3 (0.11)	5.5 (0.17)	0.19
Percentage change in SU	−2.5 (4.3)	−19.0 (2.4)	−23.6 (3.1)	< 0.001
Delta SU, mg/dl, mean (SEM)	−0.35 (0.23)	−1.4 (0.20)	−1.95 (0.26)	< 0.001
Plasma oxypurinol (umol/l) mean, (SEM)	92.8 (9.8)	151.3 (11.9)	185.1 (16.9)	< 0.001

*R+* amount of allopurinol (milligrams) above creatinine clearance-based dose, *SU* serum urate

### Adverse events

For individual liver function tests and CrCL, participants with ≥ 1 adverse event during the 12-month dose escalation phase were identified. There was no significant difference across the three R+ groups in the rates of abnormal alanine aminotransferase (ALT), aspartate aminotransferase (AST), gamma-glutamyl transferase (GGT), alkaline phosphatase (ALP) or changes in CrCL (Table 3 and Additional file 2: Figure S2). Serious adverse events by R+ dose group are outlined in Table 3. Numerically there were more participants with SAEs in the higher R+ group than in the lower R+ groups. However, this was not statistically significant (*p* = 0.44). None of the SAEs were thought to be allopurinol related.

**Table 3:** Abnormal renal and liver function and serious adverse events

	R+ ≤ 100 mg (*n* = 38)	R+ 101–200 mg (*n* = 46)	R+ > 200 mg (*n* = 48)	P value
Liver and renal function adverse events				
GGT	10 (26.3)	12 (26.1)	8 (16.7)	0.45
ALP	3 (7.9)	1 (2.2)	4 (8.3)	0.39
AST	4 (10.5)	3 (6.5)	0 (0)	0.09
ALT	3 (7.9)	4 (8.7)	8 (16.7)	0.35
CrCL increase > 20%	6 (15.8)	5 (10.9)	8 (16.7)	0.70
CrCL decrease > 20%	11 (28.9)	9 (19.6)	14 (29.2)	0.49
Serious adverse events				
	R+ ≤ 100 mg (*n* = 38)	R+ 101–200 mg (*n* = 46)	R+ > 200 mg (*n* = 48)	Total
Cardiac disorders	1	4 (4)	4 (3)	9
Gastrointestinal disorders	1	2 (2)	3 (3)	6
General disorders		1	2 (2)	3
Hepatobiliary disorders			1	1
Infections and infestations		3 (3)	3 (3)	6
Injury, poisoning and procedural complications	1	1	2 (2)	4
Investigations		1		1
Metabolism and nutrition	1			1
Musculoskeletal		3 (2)		3
Nervous system disorders	3 (2)			3
Psychiatric disorders	1			1
Renal and urinary disorders		2 (2)		2
Respiratory		1		1
Skin			1	1
Total number of events (number of participants (% of participants))	8 (4 (10.5%))	18 (8 (17.4%))	16 (10 (20.8%))	42

Renal and liver function data are reported as the number of participants with at least one CTCAE adverse event in the 12-month dose escalation phase of the study. Serious adverse events are reported as the number of events (number of participants)

*R+* amount of allopurinol (milligrams) above creatinine clearance (CrCl)-based dose, *GGT* gamma-glutamyl transferase, *ALP* alkaline phosphatase, *AST* aspartate aminotransferase, *ALT* alanine aminotransferase

There were 9 participants who died during the 12-month dose escalation phase of the study and were not included in the 132 participants used for the analysis. Details of these participants and the cause of death are outlined in Table 4. There was no significant difference in plasma oxypurinol concentration at the study visit preceding death and in the plasma oxypurinol at 12 months in the 132 participants who completed the dose escalation phase (mean (SEM) in deceased participants 176 (34.6) μmol/l vs. 147 (8.5) μmol/l on those who survived; *p* = 0.40). There were no severe cutaneous adverse reactions to allopurinol.

**Table 4:** Details of the nine participants who died during the dose escalation phase of the study

Month of death	Baseline allopurinol dose	Allopurinol dose at death	R+ (mg/day)	Baseline CrCL (ml/min)	Urate (mmol/l) at visit preceding death	Oxypurinol (μmol/l) at visit preceding death	Cause of death
11	200	400	+ 300	28	0.34	341	Heart failure
11	300	400	+ 250	32	0.37	64	Lung infection
3	200	250	+ 100	37	0.45	205	Heart failure
9	600	400	+ 150	84	0.64	0	Acute coronary syndrome
5	150	250	+ 50	65	0.37	288	Heart failure
10	300	300	+ 150	46	0.40	201	Cardiac arrest
9	200	250	+ 150	31	0.4	164	Heart failure
4	600	600	+ 400	56	0.46	179	Aortic dissection
2	300	300	+ 100	51	0.40	139	Heart failure

*R+* amount of allopurinol (milligrams) above creatinine clearance-based dose

### Factors predicting R+ group at month 12

We next explored a variety of clinical factors to determine which were independently associated with the R+ group at 12 months. Multivariate analysis indicated that calculated CrCL, baseline serum urate, weight and baseline allopurinol dose were all significantly positively associated with allopurinol dose at 12 months (*p* < 0.01 for all, *R*^2^ = 0.42). Presence of tophi, age, diuretic use and duration of gout were not associated with R+ group (Additional file 3: Table S1).

## Discussion

This analysis has shown that the majority of people with gout who do not have target serum urate on CrCL-adjusted doses require a further increase in allopurinol dose of 200 mg or less to achieve target serum urate. The multivariate analysis has identified four readily available clinical variables that are associated with requiring higher allopurinol doses; SU, CrCL, weight and allopurinol dose.

It has previously been reported that there is a ceiling effect with allopurinol, such that no matter how high the allopurinol dose there is a limit to the urate-lowering effect [8]. Consistent with this concept, our analysis has shown a flattening of the SU decrease, percentage change in SU and absolute change in serum urate once the R+ dose is > 200 mg daily (Fig. 2). However, this may in part be due to poor adherence in the higher R+ groups, noting the lower plasma oxypurinol concentrations in those failing to achieve target urate. The number of tablets required for some of the higher R+ doses may be a barrier to adherence.

While there was no increase in AEs associated with higher R+ doses, there were numerically more SAEs. These SAEs were not deemed allopurinol-related. In addition, the R+ doses of the nine individuals who died during the dose escalation phase of the study ranged across all R+ groups, and plasma oxypurinol concentrations were not significantly higher in these patients than in those who completed the study. The large numbers and types of SAEs are not surprising considering the high prevalence of co-morbidities in the study population. As anticipated, there were no cases of AHS despite the R+ doses; this is not unexpected as participants had been tolerating allopurinol for at least one month prior to study entry and the study was not powered to detect this rare event.

Of particular importance were the cardiovascular SAEs. The role of the xanthine oxidase inhibitors allopurinol and febuxostat in cardiovascular events has been the subject of much interest. The recently published study, the Cardiovascular Safety of Febuxostat and Allopurinol in Patients with Gout and Cardiovascular Morbidities, reported no significant difference between allopurinol and febuxostat in the primary composite endpoint of cardiovascular events in people with gout and established cardiovascular comorbidities at baseline. However, there was significantly increased risk of cardiac and all-cause mortality with febuxostat compared to allopurinol [9]. Of importance in this study of 6190 people, the starting dose of febuxostat was 40 mg daily with an increase to 80 mg/ daily if SU was > 6 mg/dl at week 2, the starting dose of allopurinol was dependent on kidney function and allopurinol was systematically increased to achieve target urate < 6 mg/dl with a maximum dose of 600 mg daily, reduced to 400 mg daily in those with estimated creatinine clearance of 30 to < 60 ml/min [9]. Whether there is a protective effect of allopurinol remains unclear. A recent meta-analysis of 81 randomized controlled trials reported that allopurinol was associated with a lower risk of myocardial infarction (OR 0.38, 95% CI 0.17–0.83), hypertension (OR 0.32, 95% CI 0.18–0.58), total cardiovascular events (OR 0.48, 95% CI 0.31–0.75) and serious total cardiovascular events (OR 0.56, 95% CI 0.36–0.86, *I*^2^ = 44%) compared to placebo or no treatment. In comparison, febuxostat and topiroxostat were not associated with an increased or decreased risk of cardiovascular events [10]. Interestingly, on meta-regression higher doses of allopurinol (> 300 mg/day) were associated with higher risk of total cardiovascular events [10]. In the current study 8/9 deaths were cardiovascular-related. Further studies are required to determine whether allopurinol has a protective effect against cardiovascular events and whether this is dose-related.

There are some limitations to this study. The populations in this study had a high prevalence of co-morbidities which is fairly typical for people with gout. One would anticipate that in a heathier population with gout, higher R+ does would possibly be better tolerated. In addition, this was a pre-planned secondary analysis of the main study.

## Conclusion

A wide range of R+ doses are required to achieve target SU. Four easily obtained clinical variables may be helpful in predicting higher allopurinol dose requirement. The relationship between higher R+ dose and SAEs is unclear.

## Additional files

Additional file 1: **Figure S1.** Effect of diuretic on (A) serum urate, (B) % change in serum urate, (C) delta serum urate and (D) plasma oxypurinol by R+ group at month 12 of the dose escalation phase. *p < 0.05. (JPG 560 kb)

Additional file 2: **Figure S2.** Creatinine clearance (CrCL) at 12 months by R+ group. (TIF 168 kb)

Additional file 3: **Table S1.** Multivariate analysis of factors associated with R+ group at month 12. (DOCX 14 kb)

## Abbreviations

AE: Adverse event
AHS: Allopurinol hypersensitivity syndrome
ALP: Alkaline phosphatase
ALT: Alanine aminotransferase
AST: Aspartate aminotransferase
C: Control
CI: Confidence interval
CrCL: Creatinine clearance
D: Daily
DE: Dose escalation
GGT: Gamma-glutamyl transferase
mg: Milligrams
ml/min: Millilitres per minute
OR: Odds ratio
R +: Milligrams of allopurinol above creatinine clearance-based dose
SAE: Serious adverse event
SEM: Standard error of the mean
SU: Serum urate
