# Supplementary figures and images for: How much allopurinol does it take to get to target urate? Comparison of actual dose with creatinine clearance-based dose
Source: Arthritis Res Ther. 2018 Nov 16;20:255. doi: 10.1186/s13075-018-1755-0 (PMC6240322; doi:10.1186/s13075-018-1755-0)

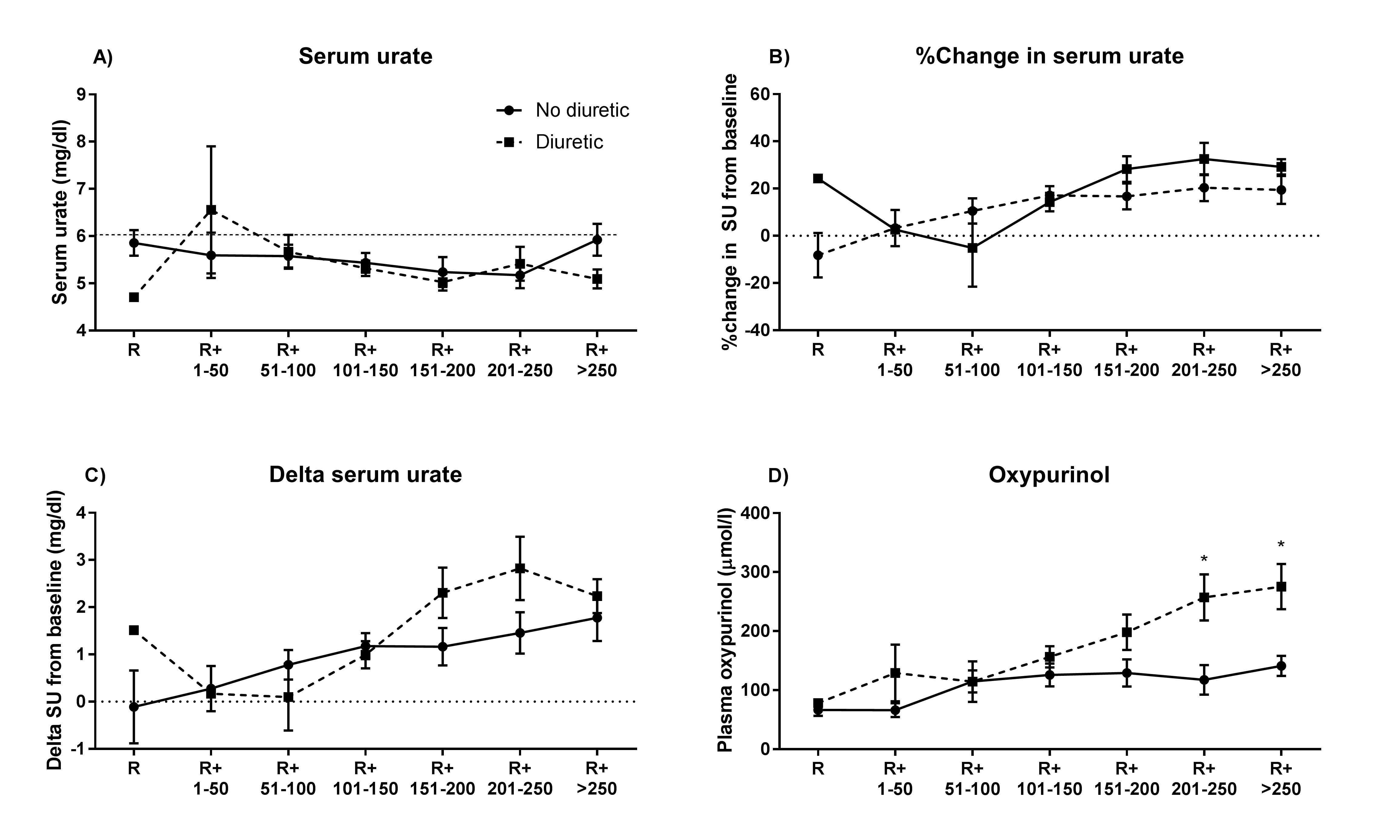

Supplement: Supplementary file 1 — Figure S1. Effect of diuretic on (A) serum urate, (B) % change in serum urate, (C) delta serum urate and (D) plasma oxypurinol by R+ group at month 12 of the dose escalation phase. *p < 0.05. (JPG 560 kb) [file 13075_2018_1755_MOESM1_ESM.jpg]

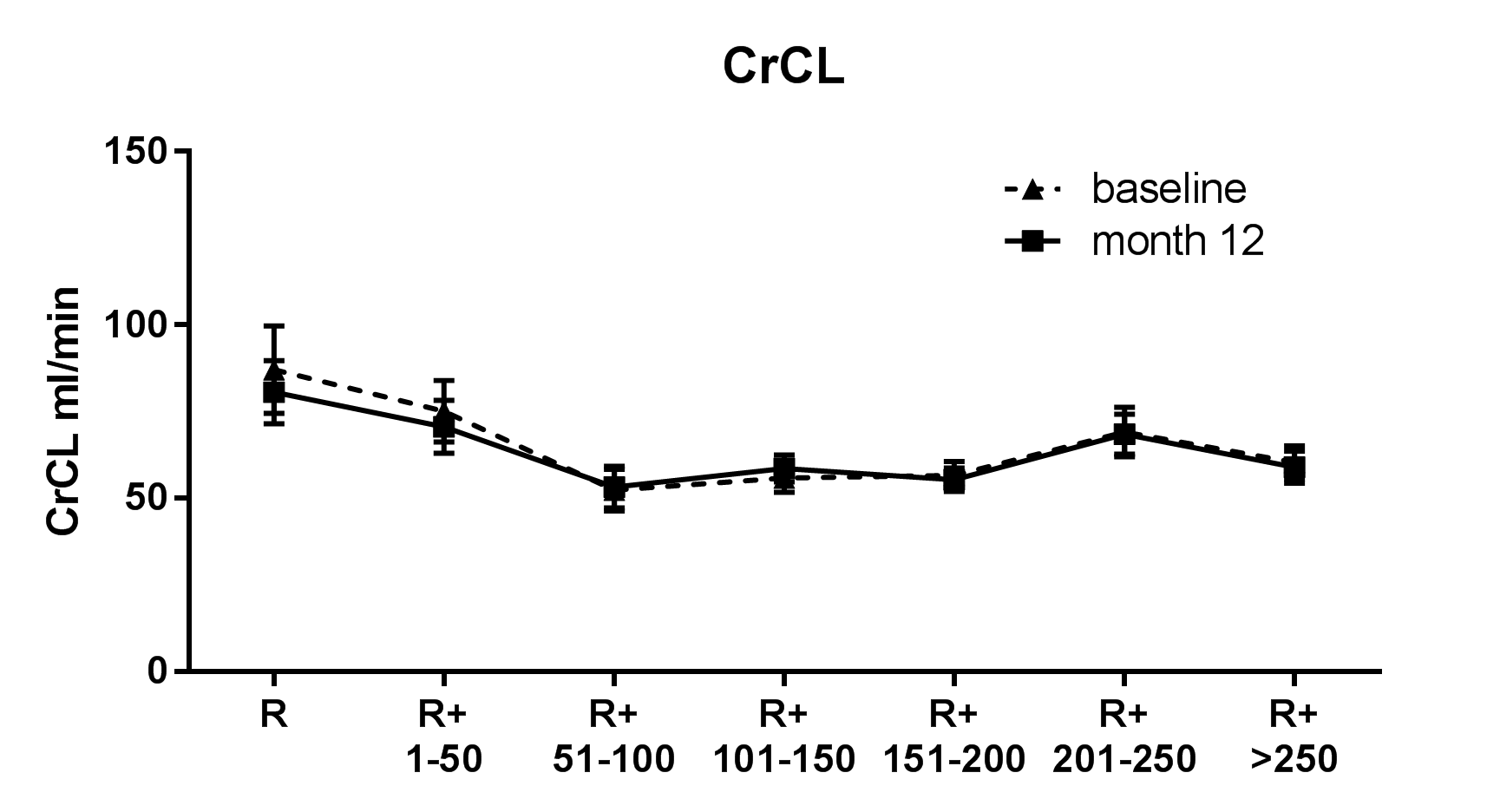

Supplement: Supplementary file 2 — Figure S2. Creatinine clearance (CrCL) at 12 months by R+ group. (TIF 168 kb) [file 13075_2018_1755_MOESM2_ESM.tif]
