# Supplementary material for: How much allopurinol does it take to get to target urate? Comparison of actual dose with creatinine clearance-based dose
Source: Arthritis Res Ther. 2018 Nov 16;20:255. doi: 10.1186/s13075-018-1755-0 (PMC6240322; doi:10.1186/s13075-018-1755-0)
Supplement: Supplementary file 3 — Table S1. Multivariate analysis of factors associated with R+ group at month 12. (DOCX 14 kb) [file 13075_2018_1755_MOESM3_ESM.docx]

**Table S1 Multivariate analysis of factors associated with R+ group at month 12**

|  | | Unstandardized Coefficients | | t | p. |
| --- | --- | --- | --- | --- | --- |
|  |  | B | Std. Error |  |  |
| Variables included in final model | (Constant) | -1.504 | 0.411 | -3.660 | 0.000 |
|  | Current allopurinol dose (mg/day) | 0.003 | 0.001 | 3.635 | 0.000 |
|  | Urate (mg/dl) | 0.191 | 0.039 | 4.925 | 0.000 |
|  | Weight (kg) | 0.009 | 0.003 | 3.191 | 0.002 |
|  | CrCl (ml/min) | 0.007 | 0.003 | 2.626 | 0.010 |
| Variables excluded from final model | Duration gout | .002^e^ | 0.027 | 0.003 | 0.991 |
|  | Age | -.089^e^ | -0.849 | -0.086 | 0.523 |
|  | Tophi present | -.071^e^ | -0.885 | -0.090 | 0.903 |
|  | On a diuretic | .047^e^ | 0.524 | 0.053 | 0.720 |
